# How Hormones and MADS-Box Transcription Factors Are Involved in Controlling Fruit Set and Parthenocarpy in Tomato

**DOI:** 10.3390/genes11121441

**Published:** 2020-11-30

**Authors:** Barbara Molesini, Valentina Dusi, Federica Pennisi, Tiziana Pandolfini

**Affiliations:** Department of Biotechnology, University of Verona, Strada Le Grazie, 15, 37134 Verona, Italy; valentina.dusi@univr.it (V.D.); federica.pennisi@univr.it (F.P.); tiziana.pandolfini@univr.it (T.P.)

**Keywords:** tomato, fruit set, parthenocarpy, phytohormones, MADS-box transcription factors

## Abstract

Fruit set is the earliest phase of fruit growth and represents the onset of ovary growth after successful fertilization. In parthenocarpy, fruit formation is less affected by environmental factors because it occurs in the absence of pollination and fertilization, making parthenocarpy a highly desired agronomic trait. Elucidating the genetic program controlling parthenocarpy, and more generally fruit set, may have important implications in agriculture, considering the need for crops to be adaptable to climate changes. Several phytohormones play an important role in the transition from flower to fruit. Further complexity emerges from functional analysis of floral homeotic genes. Some homeotic *MADS-box* genes are implicated in fruit growth and development, displaying an expression pattern commonly observed for ovary growth repressors. Here, we provide an overview of recent discoveries on the molecular regulatory gene network underlying fruit set in tomato, the model organism for fleshy fruit development due to the many genetic and genomic resources available. We describe how the genetic modification of components of this network can cause parthenocarpy, discussing the contribution of hormonal signals and MADS-box transcription factors.

## 1. Introduction

### The Transition from Flower to Fruit in Tomato

Tomato is one of the most important crops worldwide cultivated for the nutritional value of its fruit, which is a source of health-promoting compounds such as vitamins, carotenoids, phenolic compounds, and small peptides [1]. Furthermore, tomato has been adopted as an experimental model for studying fleshy fruit growth, development, and ripening. Botanically, the tomato fruit that originates from the ovary, the expanded basal portion of the pistil, is a berry composed of pericarp derived from the ovary wall, the placenta, and the pulp containing seeds [2]. Depending on the tomato cultivar, the pistil can originate either from one or more modified leaves (carpels), and the number of carpels in the pistil corresponds to the number of locules in the fruit. Studies conducted principally on model species, such as *Arabidopsis thaliana* and *Antirrhinum majus*, revealed that from flower initiation to the development of the mature flower (anthesis), the floral organs develop in concert, through a tightly controlled genetic regulation where the MADS-box transcription factor family plays a significant role [3]. Shortly before anthesis, the growth of the unpollinated ovary is actively blocked by developmental repressors and cell division temporarily stops. The control on ovary quiescence in tomato and *Arabidopsis* is exerted at least in part by negative factors derived from the communication between the anthers and the ovary [4,5]. After successful completion of pollination and ovule fertilization, the coordinated action of growth signals acts in relieving the ovary growth repression [6,7]. In contrast, in the absence of positive stimuli, pistils enter senescence and flowers abscise in a few days [8,9]. The switch from the static condition of the unpollinated ovary to that of rapidly growing fruit after fertilization is called the fruit set phase (phase I), after which fruit rapidly enlarges initially through a period of intense cell division (phase II; 1–2 weeks after fruit set). Fruit growth then continues through cell expansion (phase III) until ripening, which in tomato normally occurs ~35 days after fruit set [10]. Auxins, gibberellins (GAs), cytokinins (CKs), abscisic acid (ABA), ethylene, and brassinosteroids (BRs) have been implicated in controlling different stages of fruit growth [1,11,12], with auxins and GAs being the crucial promoting hormones of fruit initiation [5,7,13,14]. A role for auxin in the regulation of cell division and GAs in controlling cell expansion has also been proven in post-pollination fruit growth [7]. Besides changes in hormonal metabolism, alterations in photosynthesis and sugar metabolism are the major events occurring during the transition from flower to fruit [15]. MADS-box transcription factors have also emerged as one of the players recruited for the regulation of fruit set [16,17,18].

Fruit set is a very critical phase because it is more sensitive to endogenous and exogenous signals than later stages of growth [15]. Insufficient supply of nutrients, such as the phloem-imported sucrose, and adverse environmental conditions, such as drought or excessive/low temperatures, may impair the reproductive process, leading to the abortion of either flowers, seeds, or fruit with dramatic implications for fruit productivity [15,19]. The induction of parthenocarpy, which is the formation of seedless fruit in the absence of pollination and fertilization [4], could help prevent problems linked to low fruit yield under unfavorable conditions [13,20]. Parthenocarpy is generally the consequence of precocious activation of molecular events occurring normally upon pollination and fertilization. Some species or varieties (e.g., genetic mutants or plants with altered ploidy) have a natural capacity to produce parthenocarpic fruit [10,20]. In addition, parthenocarpy can be artificially obtained by applying synthetic growth factors to unpollinated ovaries or by genetic engineering [13]. Here, we review the recent discoveries on the molecular regulatory gene network underlying fruit set, and we describe how genetic modification of components of this network can cause parthenocarpy, with particular reference to the contribution of hormonal signals and MADS-box transcription factors.

## 2. Hormonal Regulation of Fruit Set

### 2.1. Auxins

The current molecular model of fruit set contemplates that before pollination and fertilization, ovary growth is blocked by a repressor complex, which is inactivated by auxin after fertilization [6]. Immediately after pollination and fertilization, auxin content (with indole-3-acetic acid—IAA—the major active auxin) increases within the ovary, activating the auxin signaling pathway that initiates fruit set. Auxin is perceived by its receptor, the TRANSPORT INHIBITOR RESPONSE 1 (TIR1), which is the F-box protein component of the E3 ubiquitin ligase complex, called Skp1/Cullin/F-box complex (SCF^TIR1^) [21]. Auxin acts as a “molecular glue”, stabilizing interaction between *At*TIR1 and its target proteins AUXIN/INDOLE-3ACETIC ACID (Aux/IAAs), thus promoting ubiquitination and degradation of the Aux/IAAs through SCF^TIR1^ and the proteasome [22,23,24]. In this way, free AUX/IAA-AUXIN-RESPONSE FACTORs (ARFs) can activate auxin response gene expression [22,23,24]. Overexpression of *SlTIR1* in tomato has resulted in several phenotypical modifications, including parthenocarpy [25]. The increased expression of *Sl*TIR1 would promote the degradation of Aux/IAA repressors with the subsequent destabilization of the AUX/IAA-ARF inhibitory complex [25]. Members of the Aux/IAA and ARF transcription factors have been demonstrated to play a role in fruit set. The tomato *entire* mutant, which carries a single base deletion in the Aux/IAA gene *SlIAA9*, exhibited parthenocarpic fruit development and alterations in leaf morphogenesis [26]. Comparable phenotypes were obtained when the expression of the *SlIAA9* gene was downregulated by RNA silencing, confirming that *Sl*IAA9 is a negative regulator of the transition from flower to fruit, besides being implicated in leaf development [27]. The auxin response factor *Sl*ARF7 is another negative regulator of fruit set because of its downregulation by RNA-silencing induced parthenocarpy [28]. *SlARF7*-silenced fruit displayed morphological alterations attributable to altered auxin and GA responses [9]. In particular, *SlARF7*-silenced fruit is heart-shaped, a trait typically observed in fruit treated with excessive auxins, and smaller in size with a thick pericarp due to increased cell expansion, an effect observed in fruit derived from GA-treated flowers [5]. The formation of an auxin signaling inhibitory complex between *Sl*IAA9 and *Sl*ARF7 has been recently demonstrated by Y2H screen and co-immunoprecipitation assays [29], thus supporting the model proposed for the action mechanism of auxin in fruit set control [6]. In addition to ARF7, the introduction of the *Arabidopsis ARF8/FRUIT WITHOUT FERTILIZATION* (*FWF*) (i.e., *Atarf8-4*) mutant allele in the tomato cultivar “Monalbo”, characterized by a moderate parthenocarpic ability, enhanced this phenotypic trait [30,31]. The role of ARF8 as a key negative regulator involved in parthenocarpic fruit development has also been demonstrated in eggplant [32]. Parthenocarpy was also observed by using the artificial microRNA strategy (amiRNA) to silence the *ARF5* gene in tomato [33]. No other evident modifications in both vegetative growth and floral morphology were observed in amiRNA*SlARF5* lines, when compared with wild-type plants [33].

Besides acting on components of the auxin signaling pathway, parthenocarpic fruit development can be achieved in tomato by increasing the content of IAA in the ovary [34,35,36,37]. Recently, a parthenocarpic eggplant mutant, named *pad-1*, was shown to accumulate a high level of IAA in the ovaries [38]. *Pad-1* gene encodes an aminotransferase, which catalyzes the reverse reaction of Tryptophan (Trp) aminotransferase. In *Arabidopsis,* Trp aminotransferase converts Trp into indole-3-pyruvic acid (IPyA), which is then transformed in IAA by members of the YUCCA family [39,40]. Thus, Pad-1 regulates the synthesis of IAA by reducing the content of the precursor IPyA. In the same work, it was proven that downregulation of the *Pad-1* orthologous genes in both tomato and pepper determined parthenocarpic fruit development [38]. In ovaries of both tomato and pepper transgenic plants, IPyA and IAA levels were greater than those in wild-type ovaries. This implies that Pad-1 is a negative regulator of fruit set acting in the maintenance of low IAA levels in the ovary [38]. A role of auxin efflux carrier PIN-formed 4 (*Sl*PIN4) in fruit set has also been demonstrated [41]. *SlPIN4* displays a strong expression in the ovary, which increases during flower development reaching a maximum in flowers at anthesis, then decreasing during fruit development. Silencing of *SlPIN4* in tomato determined the development of parthenocarpic fruit, besides morphological alterations in sepals and stamens and, rarely, an increase in the carpel numbers [41]. The observed phenotype was associated with slight modifications of auxin homeostasis at the early stages of flower bud development [41]. Thus, it has been suggested that *Sl*PIN4 acts by modifying the local distribution of auxin in the ovary and nearby tissues [41].

An elevated amount of IAA in very young flower buds (i.e., 100-fold higher than that in wild-type ones) and a reduction in polar auxin transport were observed when *AUCSIA-1* and *AUCSIA-2* (AUxin Cum Silencing Action) genes were silenced in tomato [42]. The transcript levels of *AUCSIA* genes are strongly downregulated after anthesis, a pattern commonly shown by other negative regulators of fruit set [27,28]. In transgenic plants, the silencing of *AUCSIA* gene caused parthenocarpic fruit development and other auxin-related phenotypes such as leaf fusions [42]. *AUCSIA* genes code for small peptides of 53 amino acids, and although the mechanism of action is yet unknown, it has been suggested, that they may participate in multiprotein complexes involved in auxin transport [42], because of their minimal molecular mass and the presence of a Tyr-based sorting motif involved in endocytosis [43].

Overall, these studies revealed that parthenocarpy can be achieved by manipulating auxin action at different levels, by acting on its biosynthesis, signaling cascade, and transport, corroborating the crucial role played by this hormone in the control of fruit set. In addition, these observations suggest that different ARFs have a redundant role in the control of ovary growth.

### 2.2. Gibberellins (GAs)

Successful pollination and fertilization determine the increase of active GA content in the ovary, associated with augmented expression of GA biosynthetic enzymes, such as the GA 20-oxidase, and reduction in the expression of GA catabolic enzymes, such as GA 2-oxidase [7]. The overexpression of citrus GA biosynthetic gene *GA 20-oxidase 1* (*GA20ox1*) in tomato induced parthenocarpic fruit growth linked to an increased content of GA_4_ [44]. The flowers of *GA20ox1*-overexpressing plants displayed alterations in pistil development with a long style protruding from the flower, thus preventing self-pollination at the stigma surface [44]. Silencing of the genes encoding for the GA inactivating enzyme, *GA2-oxidase* (*GA2ox*), which in tomato constitute a small multigenic family of five members, caused an accumulation of active GAs in the ovary and parthenocarpic fruit development [45]. As observed for auxin signaling, it was demonstrated that also the GA signal transduction pathway is blocked in the ovary before fertilization by the presence of a transcriptional repressor, a DELLA protein [46]. The tomato genome presents only one *DELLA* gene (*PROCERA/SlDELLA*) and a mutation of this gene (i.e., the *procera* mutant) [47,48] caused a phenotype referable to a constitutive GA response, which consisted of elongated internodes, altered branching architecture, thinner leaves, and reduced leaf complexity [49]. An in-depth characterization of the *procera* mutant revealed alterations also in flower morphology [50]. Besides this, the ovaries from *procera* flowers showed a very strong parthenocarpic capacity [50]. Furthermore, the release of DELLA repression in tomato obtained by RNA silencing, allowed parthenocarpic fruit growth [46]. Parthenocarpic *DELLA*-silenced fruit were smaller than wild-type, had an elongated morphology and a reduction in the pericarp cell number [46]. In fact, in *SlDELLA*-silenced parthenocarpic fruit, auxin-regulated cell division (phase II) is bypassed, while cell expansion phase III is activated [46]. *DELLA*-silenced tomato flowers displayed various alterations, such as shorter anther filaments, longer styles with stigma protruding from the anthers, and altered stigma morphology, suggesting the involvement of DELLA also in regulation of sexual organ growth [46].

Considering that manipulation of GA signaling leads to formation of parthenocarpic fruit elongated in shape and smaller than seeded fruit, whereas parthenocarpic fruit obtained by altering IAA signaling is generally similar in size and shape to seeded fruit [27,28,31], auxin has been recognized as an early signal acting upstream from GA responses in fruit initiation [6]. The crosstalk between auxin and GA signaling components in fruit set regulation has recently been demonstrated [29]. It was reported that *Sl*ARF7 can directly interact with both *Sl*IAA9 and *Sl*DELLA through distinct protein regions. *Sl*DELLA and *Sl*ARF7/*Sl*IAA9 play opposite roles in the feedback regulation of genes involved in GA and auxin metabolism. On the other hand, *Sl*DELLA and *Sl*ARF7/*Sl*IAA9 act additively in the regulation of ovary/fruit growth-related genes [29].

### 2.3. Cytokinins (CKs)

Like auxins and GAs, CKs function as an endogenous signal for fruit set and growth [10]. Two distinct peaks of CK accumulation have been detected in tomato [51]. The first peak appears in unpollinated ovaries at anthesis and is due to transient accumulation of CK ribosides and isopentenyladenine. The second peak occurs after fertilization and results from accumulation of CK trans-zeatin [51]. As CKs regulate cell division, the first peak is associated with the initial growth of the ovary in unpollinated flowers until it reaches a mature size, whereas the second peak is linked to the stimulation of cell division in the phase II of fruit growth [51]. The accumulation of CKs is due to up-regulation in expression of corresponding biosynthetic genes [51]. However, to our knowledge, none of the genes implicated in CK biosynthesis and/or action has been manipulated by genetic engineering to obtain seedless parthenocarpic fruit. Thus, evidence of the role of CKs in fruit initiation is based only on experiments using exogenous application on flowers. Synthetic CK *N*-(2-chloro-pyridin-4-yl)-*N*’-phenylurea (CPPU) and trans-zeatin sprayed on unfertilized flowers induced parthenocarpy in tomato, with CPPU being the most effective [51,52]. In terms of pericarp thickness, the effect of CPPU on parthenocarpic fruit development was comparable to that of GA_3_ application [52]. However, when an inhibitor of GA biosynthesis (i.e., paclobutrazol (PCB)) was applied concomitantly with CPPU to unpollinated ovaries, the effect of CPPU on ovary growth was lower than that of GA_3_, suggesting that CPPU-induced parthenocarpy is partly dependent on GA accumulation [52]. CPPU-induced parthenocarpic fruit showed enhanced accumulation of GA and IAA, due to an increased expression of related biosynthetic enzymes [52]. Thus, parthenocarpy induced by CKs occurs partially through modulation of GA and IAA metabolism [52].

### 2.4. Other Hormones

Data from literature also indicates that the phytohormones brassinosteroids (BRs), ethylene, and abscisic acid (ABA) are also implicated in fruit set and growth.

BRs are a group of steroidal hormones that regulate several aspects of plant growth and development, also through the synergistic interaction with auxins [53]. BR biosynthesis is induced in developing seeds and fruit of several species, including tomato [54,55,56]. In agriculture, BRs are used to promote fruit crop ripening and productivity [57,58]. Spraying BRs (24-epibrassinolide (EBR)) on grape flowers after blooming and at veraison increased yield, while the application of EBR before budbreak and pre-bloom had no effect [59]. The application of BRs stimulated ripening in tomato pericarp discs, grape, and strawberry [60,61,62]. The effect of BRs on ripening of climacteric fruit was associated with an increase in ethylene production [60].

Until now, a role for BRs in fruit set has been demonstrated only in cucumber, where EBR applied to unpollinated ovaries of a non-parthenocarpic cultivar induced parthenocarpy, while applying an inhibitor of BR biosynthesis (i.e., brassinazole) to the flowers of a parthenocarpic cultivar caused a reduction in fruit-set capacity [55]. Parthenocarpic fruit derived from EBR-treated flowers appeared similar in length to those derived from pollinated flowers [55]. The expression of cell cycle-related genes (i.e., cyclins and cyclin-dependent kinases) was induced by both pollination and EBR treatment, indicating a role for BRs in promoting cell division in the ovary [55]. BRs seem not to be involved in tomato fruit set, because the exogenous application of EBR to flowers of the tomato Micro-Tom cultivar, which carries a mutation in the DWARF locus responsible for BR biosynthesis, was unable to induce parthenocarpy [63,64].

The gaseous hormone, ethylene, is involved in several aspects of reproductive development, such as senescence/abscission of floral organs, and fruit ripening [14]. Several transcriptomic studies conducted on tomato ovaries during fruit set, revealed that after pollination the transcription of genes implicated in ethylene biosynthesis and signaling decreased when compared with levels in unpollinated ovaries [17,65]. The level of ethylene produced in unpollinated and pollinated pistils of wild-type plants has been quantified in [66]. Ethylene synthesized in unpollinated wild-type pistils remained high, whereas the level of ethylene in pollinated pistils gradually declined after anthesis in conjunction with an increase in ovary/fruit diameter [66]. The application of ethylene precursor 1-Aminocyclopropane 1-carboxylic acid (ACC) to pollinated wild-type ovaries reduced fruit set efficiency compared with mock-treated ovaries [66]. In contrast, preventing ethylene perception either by treating emasculated wild-type flowers with ethylene inhibitor 1-methylcyclopropene (1-MCP) or by a mutation in the ethylene receptor *ERT1* gene (i.e., *Sletr1-1* mutant), led to parthenocarpic fruit with elongated shape due to increased cell expansion [66,67]. The ovaries of *Sletr1-1* mutant flowers contained a high level of GAs, most likely due to the increased expression of GA biosynthetic genes [66]. The application of the GA biosynthesis inhibitor, PCB, severely reduced parthenocarpic fruit set, observed in 1-MCP treated wild-type and ethylene-insensitive *Sletr1-1* mutant plants, demonstrating that GA biosynthesis is necessary to induce parthenocarpy by blocking ethylene action [66]. In this regard, ethylene could exert its inhibitory role on ovary growth by stabilizing DELLA repressors as observed in *Arabidopsis* [66,68]. Parthenocarpic fruit development has been obtained in tomato overexpressing the *SlTPR1* gene, which codes for a tetratricopeptide repeat protein able to interact with ethylene receptors in a yeast two-hybrid analysis and in vitro [69]. The overexpression of *SlTPR1* was proposed to result in a constitutive ethylene response [69]. In this regard, *Sl*TPR1 would compete with CTR1, a negative regulator of the ethylene response [70,71], for the binding to ethylene receptors. *Sl*TPR1 overexpression did not result in increased ethylene production but affected the expression of some ethylene- and auxin-responsive genes [69]. In particular, *35S::SlTPR1* parthenocarpic tomato plants exhibited a reduced expression of *SlIAA9* gene [27,69]. Thus, *Sl*TPR1, besides playing a role in ethylene signaling, is involved in the crosstalk between ethylene signaling and auxin responses during fruit set [69].

ABA is a phytohormone principally involved in the regulation of stress responses, seed and bud dormancy, as well as in the differentiation of floral organs, fruit development, and ripening [72,73]. Data from transcriptomic analyses revealed that genes involved in ABA biosynthesis and response were highly expressed in unpollinated mature tomato ovaries and their expression decreased after fruit set, also supporting a role for this hormone during transition from flower to fruit [17,65]. However, the application of inhibitors of ABA biosynthesis to unpollinated ovaries did not result in fruit set induction, and no inhibitory effect on fruit set has been observed when applying ABA to pollinated ovaries [74]. This indicates that rather than just ABA concentration, it may be the balance between ABA and other hormones which regulates fruit set [74]. The *SlNCED1* gene codes for 9-cis-epoxycarotenoid dioxygenase, the principal biosynthetic enzyme responsible for ABA levels in tomato ovaries [75]; its expression decreases during fruit set induced by synthetic auxin and GA_3_ treatments [74]. Based on these findings, it has been suggested that the role of ABA during fruit set is to inhibit the growth of the ovary maintaining the pistil in its dormant state, thus acting as the antagonist of the promoting roles played by IAA and GAs [65,74]. Recently, a study depicted a more complex scenario for *Sl*NCED1 action in pistil development and fruit set [75]. In this work, the overexpression in tomato of *SlNCED1* caused an increase in ABA levels in the pistils and caused phenotypical alterations in ovary morphology and styles [75]. Comparisons of the transverse and longitudinal diameters of the ovary before and after full bloom, indicated that the onset of ovary growth in *SlNCED1*-overexpressing lines started before anthesis, suggesting that the ovary growth constriction is released before pollination. Ninety percent of *SlNCED1*-overexpressing fruit was parthenocarpic, but the overexpression of *SlNCED1* led to drastically reduced fruit set capacity (i.e., <10%) in comparison to wild-type plants [75]. Comparative transcriptomic analyses conducted on *SlNCED1*-overexpressing and wild-type pre-anthesis flower buds revealed that *SlNCED1* overexpression determined a general alteration in the expression of genes related to the action of several phytohormones including ABA, ethylene, auxin, CKs, and GAs [75].

## 3. Parthenocarpy and MADS-box Transcription Factors

Early studies on genetic control of floral development in *A. thaliana* and *A. majus* led to the discovery that MADS-box transcription factors are key regulatory genes in defining the identity of floral organs [76,77]. The functions of these homeotic genes were generally elucidated through molecular analyses of mutant plants with aberrant flower development [78]. These seminal works led to the formulation of the ABC model of flower development, which identified the homeotic genes involved in determining the identity of the organs in the four concentric whorls of the flower: sepal, petal, stamen, and carpel. The original ABC model was successively modified including D and E homeotic genes and the interaction between ABCDE proteins was described in the floral quartet model [3]. The first approach used to elucidate flower development in tomato was the identification of the orthologs of *A. thaliana* and *A. majus* MADS-box genes and the subsequent analysis of the effects produced on flower structure by their downregulation or overexpression (Table 1).

For instance, a seminal study [79] described the effects of downregulation and the ectopic expression of *Tomato Agamous 1* (*TAG1*), an ortholog of the C type *Agamous* gene of *A. thaliana*. The silencing of *TAG1* gene, which is expressed in the third and fourth whorl, resulted in abnormal development of stamens and carpels as expected, while its ectopic expression besides modifying the identity of sepals, caused male and female sterility and production of parthenocarpic fruit [79]. On the other hand, studies on natural tomato parthenocarpic mutants demonstrated a link between the development of fruit in the absence of fertilization and flower homeotic modifications [90,91]. For instance, the parthenocarpic fruit (*pat*) mutant presents aberrant androecia and ovules and consequently reduced male and female fertility [92]. In the parthenocarpic *IAA9* mutant, homeotic transformation of stamens into carpelloid features and fusion of sepals at the insertion in the receptacle were observed [93]. In this case, since *IAA9* is a repressor of auxin action, the homeotic changes observed in the mutant flowers might be an indirect consequence of the perturbation of auxin signaling. Indeed, the transcriptomic analysis carried out on tomato parthenocarpic plants obtained by silencing the *IAA9* gene [27], revealed that the expression of two MADS-box gene, *TAG1* and *Tomato Agamous-like 6* (*TAGL6*), was downregulated in the ovaries at anthesis compared to the wild-type [17]. In addition, in the wild-type ovaries, the *TAG1* and *TAGL6* transcript levels were drastically reduced after fertilization [17], a typical pattern of expression of ovary growth repressors (e.g., AUCSIA, ARF7, and PIN4) [28,41,42]. Interesting observations about the expression pattern of MADS-box genes were previously reported [94]. In this work, seven MADS-box genes, including *TAG1*, that were expressed not only during flower development but also during the first stages of fruit and seed development, were identified [94]. The authors suggested that MADS-box genes may have a role in coordinating flower and fruit/seed development, thus depicting fruit initiation and successive phases of fruit growth as a continuation of the floral developmental program [94].

The important and complex role of members of different classes of MADS-box proteins in tomato fruit initiation has emerged in several later works (Table 1). After the study described the functional characterization of TAG1 [79], another member of the C class of homeotic genes, *Tomato Agamous-like* 1 (*TAGL1*) was associated with the regulation of fruit set. The effects of the downregulation and overexpression of *TAGL1* on tomato flower and fruit development have been reported in [80]. In the *TAGL1*-silenced plants fruit ripening was inhibited, while ectopic expression of *TAGL1* resulted in varying degrees of conversion of petals into stamenoid structures, fleshy sepals, and production of seedless fruit, phenotypical alterations similar to those observed with the ectopic expression of *TAG1* [79]. The functional characterization of *Arlequin* (*Alq*), a semi-dominant T-DNA tomato mutant, further elucidated the role of *TAGL1* in fruit development [81,82,91]. In *Alq*, the T-DNA insertion in *TAGL1* leads to increased expression of *TAGL1* causing a severely altered fruit phenotype characterized by succulent sepals that follow a ripening pattern similar to that of normal fruit [81]. A recent re-examination of *Alq-TAGL1* mutant phenotype confirmed that *TAGL1* is involved in the regulation of the first steps of fruit growth as the mutant plants showed precocious fruit set and seedlessness [82]. In this mutant, the growth of the ovary prior to fertilization appeared associated with increased endogenous CKs and reduced ABA levels. Interestingly, this tendency to develop parthenocarpic fruit was not associated with loss of pollen viability [82]. These studies demonstrated the regulatory role of *TAGL1* in the transition from flower to fruit, being its correct pattern of expression in the carpel, necessary to maintain the ovary in a repressive state until fertilization.

Several lines of evidence have associated parthenocarpy in tomato with the alteration of members of the class B of tomato MADS-box genes. Petal and stamen identity in tomato are controlled by the duplicated orthologs of *A. majus DEFICIENS* (*euAP3/TAP3* and *TM6*) and *GLOBOSA* (*SlGLO1* and *SlGLO2*) lineage [84]. The analysis of tomato *APETALA3* (*TAP3*) homozygous mutant plants demonstrated that *TAP3* suppression caused the conversion of petals into stamens and stamens into carpelloid structures [95]. The downregulation of *TAP3* in the ovary obtained by the expression of a silencing construct under the control of a flower/fruit specific promoter (*P119*), resulted in partial conversion of stamens into carpelloid structures, male sterility, and parthenocarpic development of the fruit [83]. Because *TAP3*-silenced plants produce seeded fruit when manually pollinated with wild-type pollen, the authors suggested that the parthenocarpic trait is probably due to pollen impairment. In this regard, Medina and co-workers (2013) demonstrated that the early ablation of anthers as well as the transgenic inactivation of pollen development induce production of seedless fruit [96]. The parthenocarpic development of the fruit in *TAP3*-downregulated ovaries was associated with an increased GA level, leading the authors to formulate the hypothesis that stamen development negatively regulates fruit set by repressing GA biosynthesis [83]. Interestingly, the simultaneous downregulation of *SlGLO1* and *SlGLO2* by RNA silencing caused floral organ conversions that resemble those obtained by *TAP3* silencing: the carpelloid structures, that often fuse with the central gynoecium, give rise to ripe fruit structures that do not contain seeds [84]. A gene related to the *AGAMOUS* family and classified as a D member, *SlAGL11*, was demonstrated to be possibly involved in seed development [85]. When overexpressed, *SlAGL11* caused no visible modifications in vegetative growth, but alterations in floral organs. The sepals were converted into fleshy organs and started a ripening process, phenotypic modifications similar to those observed with ectopic expression of the C type *TAG1* and *TAGL1* genes [79,80]. The fruit obtained from *SlAGL11* overexpressing plants were seedless or contained few seeds and pollen was not viable [85].

A link between a MADS-box protein of the class E, the *Tomato MADS-BOX29* (*TM29*), and fruit initiation has been demonstrated [16]. *TM29* belongs to the SEPALLATA family and shows 68%, 63%, and 58% identity with *Arabidopsis* proteins SEP1 (AGL2), SEP2 (AGL4), and SEP3 (AGL9), respectively. The *TM29* transcript level is high in the primordia of all four floral organs, and in the stamen and pistil of flower buds before and during anthesis [16]. The downregulation of *TM29* caused changes in petals and stamens that appeared to be green. Furthermore, transgenic stamens tend to separate from each other forming a loose cone, and do not produce pollen. The ovaries of *TM29*-downregulated plants are larger than those of wild-type and develop in the absence of pollination, even when cross-pollinated with wild-type pollen, suggesting that *TM29* acts before ovule fertilization as a repressor of ovary growth [16].

Another example of MADS-box gene involvement in regulating fruit set came from studies on *Tomato MADS 8* (*TM8*), which was classified as an ‘early’ flowering gene, since it is mainly expressed in floral meristems and to a lesser extent in the mature flower. *TM8* overexpression produced visible modifications in the third whorl, like splayed stamen and poorly viable pollen [86]. Its expression in the form of a dominant chimeric repressor (*35S::TM8:SRDX*) produced flowers with anomalies in the fourth whorl while stamen appeared normal and pollen viable. The *35S::TM8:SRDX* plants displayed ovaries with an oblong shape and fruit devoid of seeds [86]. In the ovaries of these transgenic plants, the expression of two *AGAMOUS* genes, *TAG1* and *TAGL1*, was significantly reduced. Notably, the parthenocarpic trait had sometimes been observed by Lifschitz et al. (1993) in *TM8*-antisense tomato plants [87].

Recently, a very interesting work identified a mutant tomato plant, that displayed facultative parthenocarpy under heat stress conditions without pleiotropic effects on vegetative and reproductive development [88]. The mutant produces seedless fruit of a normal weight and shape and with viable pollen. RNA-seq and CRISPR/Cas9 gene knockout technology was applied to demonstrate that the parthenocarpic phenotype was due to a mutation in *SlAGAMOUS-LIKE 6* (*SlAGL6*). *SlAGL6* encodes a MADS-box protein of type II lineage MIKCC, subfamily *AGL6* [97]. *SlAGL6* is a good candidate to act as a factor that maintains ovary growth in an arrested phase until fertilization occurs. This is consistent with its expression pattern, which is characterized by a high expression in flower buds at anthesis and a sharp decline after fertilization. The absence of alterations in floral organs and precocious ovary growth displayed by *SlAGL6*-knockdown plants, suggests a very specific role for this gene in fruit set [97]. A similar parthenocarpic phenotype was observed in the tomato *pat-k* mutant [89]. The mutation consists of a retrotransposon insertion in the *SlAGL6* gene causing a drastic downregulation of its expression in the ovaries [89].

**Figure 1:**
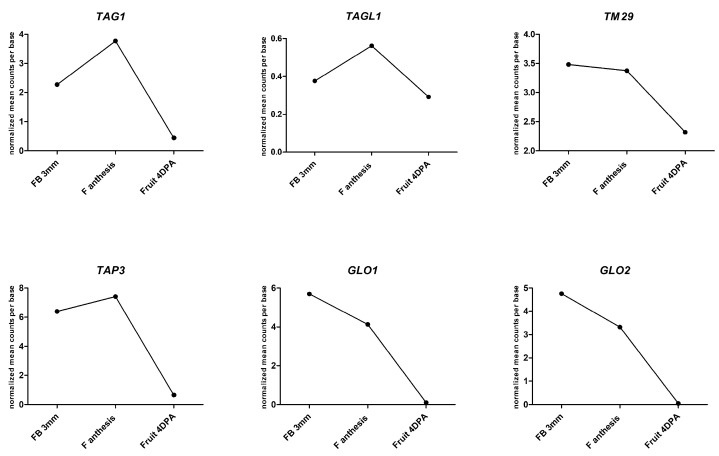
Expression level of *TAG1*, *TAGL1*, *TM29*, *TAP3*, *GLO1*, and *GLO2* genes in 3 mm-long flower buds (FB 3 mm), flowers at anthesis (F anthesis) and fruit at 4 days after anthesis (Fruit 4 dpa) of tomato Micro-Tom cultivar. Data obtained from the TomExpress database [101].

From all these studies, the involvement of MADS-box transcription factors in the regulation of fruit set and development appears to be evident, even if it is very difficult to place the information present in the literature in a clear picture. Seedlessness has been reported to occur both as a consequence of either suppression (*AGL6*, *TM8*, *TM29*, *TAP3*, *SlGLO1,* and *SlGLO2*) or ectopic overexpression (*AGL11*, *TAG1*, and *TAGL1*) of MADS-box genes. In the first case, the activity of the gene would directly or indirectly be linked either to the repression of fruit growth prior to fertilization or to maintenance of ovule or pollen viability. In the case of ectopic overexpression, parthenocarpy could represent a pleiotropic effect that highlights the need for strict spatial localization of MADS-box expression to avoid an untimely onset of ovary growth. It is interesting to note that in some cases, fruit seedlessness is accompanied by male sterility (*TAG1*, *TAGL1*, *AGL11*, *TAP3*, *TM29*, *SlGLO1*, and *SlGLO2*). It is known that early anther ablation can favour parthenocarpy, thus relieving the ovary growth repression probably by increasing GA concentration [83]. Another possible explanation is that the defective pollen fails to fertilize ovules but still produces some signals that induce fruit initiation. On the other hand, when parthenocarpy is obligatory, as in the case of *TM29* downregulation, parthenocarpic trait is most likely linked to alteration in the female organ rather than pollen defects. In this regard, an intriguing relationship between parthenocarpy and male and female gametogenesis is illustrated in two recent papers. The tomato parthenocarpic mutant called *hydra*, characterized by the absence of both male and female sporocyte development, has been described in [98]. The *HYDRA* gene is the orthologue of *SPOROCYTELESS/NOZZLE* (*SPL/NZZ*) of *Arabidopsis* and contains in its 3′UTR region the CArG box, which is a *cis* acting element for MADS-box transcription factor binding [99]. A second mutant of tomato, *sexual sterility* (*Slses*) carrying a 13bp deletion in the first exon of a *SPL*/*NZZ* homolog, exhibited incomplete ovules and sterile anthers. This mutant occasionally produces seedless fruit with a reduced size and weight compared with that of wild-type [100]. The most interesting case of MADS-box-related parthenocarpy is that of *AGL6* whose suppression results in facultative parthenocarpy, as the transgenic plants produce seeded fruit when pollinated and seedless fruit under unfavorable conditions. Both pollen and ovules are viable and no pleiotropic effects on reproductive or vegetative development, except parthenocarpy, are observed in the mutated plants [88]. Thus, differently from other MADS-box transcription factors, the role of AGL6 appeared to be exclusively related to the repression of ovary growth before fertilization [88]. The *AGL6* transcript level in the ovary reaches a peak when ovary growth is arrested just prior to fertilization and declines in young fruit at 4 days after anthesis (dpa) [88]. In the transition from flower to fruit set, a similar decline in the transcript level can also be observed for *TAG1*, *TAGL1*, *TM29*, *TAP3*, *GLO1*, and *GLO2* (Figure 1).

AGL6 may have undergone neo-functionalization, acquiring a specific role in arresting ovary growth before fertilization [88]. On the other hand, other MADS-box genes seem to have maintained, together with their homeotic function in flower development, the capacity to inhibit the growth of the ovary or to retain it in a repressive state. This redundancy in fruit set regulation may be explained by the need to strictly coordinate fruit and embryo/seed development.

## 4. Concluding Remarks

**Figure 2:**
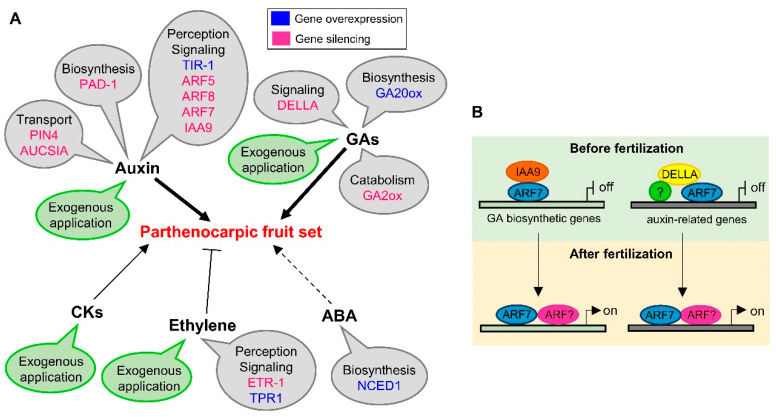
Schematic model of hormonal regulation of parthenocarpic fruit set in tomato (**A**) Parthenocarpy obtained either by exogenous treatments or by genetic manipulations of phytohormones. Gene name in blue means that the gene overexpression leads to parthenocarpy; gene name in pink indicates that the gene silencing causes parthenocarpy. TIR-1 (TRANSPORT INHIBITOR RESPONSE 1), auxin receptor; ARF5/7/8 (Auxin Response Factor 5/7/8) components of the auxin signaling pathway; PAD-1 (aminotransferase) implicated in auxin biosynthesis; PIN4 (PIN-formed 4 auxin efflux carrier), component of the auxin transport; AUCSIA (AUxin Cum Silencing Action), probably implicated in the regulation of auxin transport; DELLA, repressors of GA responses; GA20ox (GA 20 oxidase) a GA biosynthetic gene; GA2ox (GA 2 oxidase), a GA catabolic enzyme; NCED (nine-cis-epoxycarotenoid dioxygenase) an ABA biosynthetic gene; ETR-1 (Ethylene receptor 1), implicated in ethylene perception; TPR1, (tetratricopeptide repeat protein 1), able to bind the ethylene receptor. (**B**) Interplay between auxin and GAs. Before fertilization, the regulatory complex formed by DELLA, an unidentified transcriptional regulator (?), and ARF7, represses the transcription of auxin-related genes and the complex IAA9/ARF7 inhibits the transcription of enzymes involved in GAs biosynthesis. After fertilization, the increased auxin level inside the ovules determines the degradation of IAA9, thus permitting the activation of GA biosynthesis. As a consequence, DELLA repressor is degraded, allowing the dimerization of ARF7 with additional ARF (ARF?) to modulate the transcription of auxin-related genes, thus inducing ovary growth.

The transition from flower to fruit development, normally occurring after double fertilization and consequent formation of the zygote, is a crucial phase in the plant life cycle, involving an extensive reprogramming at the molecular level. The regulation of this transition suggests an integration of endogenous signals from sporophytic and gametophytic tissues and environmental cues. Successful initiation of fruit and embryo/seed development largely affects crop productivity. Fruit development can be uncoupled from embryo/seed development, giving rise to the production of seedless fruit through parthenocarpy. Parthenocarpy is a phenotypic trait that can be exploited in agricultural practice to obtain precocious fruit production under adverse environmental conditions for pollen production. In addition, the absence of seeds in some fruits is appreciated by consumers because it improves fruit quality and can be advantageous for industrial fruit processing (for instance in industrial production of fruit paste or juice). Thus, deciphering the genetic network underlying parthenocarpy, and more generally fruit set, can have important implications in agriculture, also considering the compelling need to obtain cultivars able to cope with expected changes in climatic conditions. Phytohormones are important endogenous regulators of this phase transition, as has been demonstrated by numerous studies (Figure 2, panels A and B).

Auxins and GAs have emerged as the most important players in fruit set regulation, besides their crucial role in a plethora of vegetative development processes. Many aspects of auxin and GA modes of action have been elucidated, however future research could be useful, both to study the function of other hormones as well as to unravel hormonal crosstalk. A further layer of complexity in fruit set regulation emerges analyzing the effects obtained by genetically manipulating floral homeotic genes. Some of these homeotic MADS-box genes exhibit an expression profile generally observed for repressors of ovary growth and pleiotropic activities in fruit growth and development. The *AGL6* mutation, that confers parthenocarpic fruit development in tomato without pleiotropic effects on flower development, suggests that some MADS-box genes might have undergone sub functionalization, thus conserving only the activity as ovary growth regulator, while their function in flower organ identity would have been lost. It would be interesting to deepen the research of MADS-box genes in fruit set, identifying downstream targets and elucidating the relationship between hormone signaling and MADS-box activity. The observation that some elements of the genetic network controlling the formation of flower organs and gametogenesis might also be involved in the successive phases of fruit formation and growth, supports the idea that the two developmental programs are tightly connected.

## Figures and Tables

**Table 1:** MADS-box genes involved in production of fruit in the absence of fertilization.

Gene Name Locus (id)	Class of Homeotic Genes	Genetic Modification	Male Fertility	Female Fertility	Other Alterations in Reproductive Organs	Reference
*TAG1* (Solyc02g071730)	C	Overexpression	No	No	Homeotic changes in 1st and 2nd whorl; succulent sepals	[79]
*TAGL1* (Solyc07g055920)	C PLENA subfamily	Overexpression	Yes (*Alq-TAGL1*)/No (*35S:TAGL1*)	Yes (*Alq-TAGL1*)	Succulent sepals	[80,81,82]
*TAP3* (*APETALA3*) (Solyc04g081000)	B APETALA3/PISTILLATA (AP3/PI) subfamily	Downregulation in the ovary	No	Yes	Homeotic changes in the 3rd whorl; stamen with carpelloid appearance	[83]
*SlGLO1* (Solyc08g067230.4.1) *SlGLO2* (Solyc06g059970)	B APETALA3/PISTILLATA (AP3/PI) subfamily	Downregulation of both genes	Nd *	Nd *	Homeotic changes in 2nd and 3rd whorl: petal to sepal, stamen to carpel	[84]
*AGL11* (Solyc11g028020)	D	Overexpression	No	Yes	Succulent sepals	[85]
*TM29* (Solyc02g089200)	E SEPALLATA subfamily	Downregulation	No	No	Changes in 2nd and 3rd whorl; bigger ovary	[16]
*TM8* (Solyc03g019710.3.1)	type II lineage MIKCC	Dominant repressor Antisense	Yes	Nd *	Changes in the 4th whorl; oblong ovary	[86,87]
*AGL6* (Solyc01g093960)	type II lineage MIKCC	Suppression/downregulation	Yes	Yes	No	[88,89]

Nd *: not defined.
